# Responses to Salt Stress of the Interspecific Hybrid *Solanum insanum* × *Solanum melongena* and Its Parental Species

**DOI:** 10.3390/plants12020295

**Published:** 2023-01-08

**Authors:** Neus Ortega-Albero, Sara González-Orenga, Oscar Vicente, Adrián Rodríguez-Burruezo, Ana Fita

**Affiliations:** 1 Institute for the Conservation and Improvement of Valencian Agrodiversity (COMAV), Universitat Politècnica de València, Camino de Vera S/N, 46022 Valencia, Spain; 2 Department of Plant Biology and Soil Science, Faculty of Biology, Universidad de Vigo, Campus Lagoas-Marcosendre, 36310 Vigo, Spain

**Keywords:** eggplant, heterosis, ion homeostasis, osmolyte, oxidative stress, proline, salinity, tolerance

## Abstract

Soil salinity is becoming one of the most critical problems for agriculture in the current climate change scenario. Growth parameters, such as plant height, root length and fresh weight, and several biochemical stress markers (chlorophylls, total flavonoids and proline), have been determined in young plants of *Solanum melongena,* its wild relative *Solanum insanum*, and their interspecific hybrid, grown in the presence of 200 and 400 mM of NaCl, and in adult plants in the long-term presence of 80 mM of NaCl, in order to assess their responses to salt stress. Cultivated eggplant showed a relatively high salt tolerance, compared to most common crops, primarily based on the control of ion transport and osmolyte biosynthesis. *S. insanum* exhibited some specific responses, such as the salt-induced increase in leaf K^+^ contents (653.8 μmol g^−1^ dry weight) compared to *S. melongena* (403 μmol g^−1^ dry weight) at 400 mM of NaCl. Although there were no substantial differences in growth in the presence of salt, biochemical evidence of a better response to salt stress of the wild relative was detected, such as a higher proline content. The hybrid showed higher tolerance than either of the parents with better growth parameters, such as plant height increment (7.3 cm) and fresh weight (240.4% root fresh weight and 113.3% shoot fresh weight) at intermediate levels of salt stress. For most biochemical variables, the hybrid showed an intermediate behaviour between the two parent species, but for proline it was closer to *S. insanum* (ca. 2200 μmol g^−1^ dry weight at 200 mM NaCl). These results show the possibility of developing new salt tolerance varieties in eggplant by introducing genes from *S. insanum*.

## 1. Introduction

Global food production should increase by 50% by 2050 to feed a population that will rise to 10 × 10^9^ people [1]. However, soil salinity affects over 109 × 10^6^ ha of cropland worldwide, reducing yields in more than 50% of the surface of the most productive agricultural areas—those cultivated under irrigation in arid and semi-arid regions [2,3,4,5,6]. The source of high salt concentration is primarily the presence of salt in irrigation water and the accumulation of Na^+^ and Cl^−^ in soils [2].

The effects of soil salinity on plants vary widely depending on multiple factors, but salt tolerance is mainly controlled by genotype [7,8]. An operational threshold has been established to classify species into salt-sensitive (glycophytes) and salt-tolerant (halophytes), as well as those able to complete their life cycle under soil salinities equivalent to more than 200 mM NaCl [9,10,11]. Different species have developed protective mechanisms to cope with salt stress, from the physiological and biochemical to the molecular level, involving complex networks of genes, proteins and metabolites [12].

Salinity induces detrimental effects in glycophytes, such as (i) reduced water availability, (ii) ion toxicity, (iii) oxidative stress and (iv) K^+^ deficiency [13,14], which lead to a reduction in plant growth and biomass accumulation, and eventually, plant death [15,16].

Salt stress alters the normal metabolic processes of the plant: photosynthesis and energy production, lipid metabolism, nutrient acquisition, the integrity of cellular membranes and the activity of enzymes [17]. Plant growth under such stressful conditions depends on the efficiency of the mechanisms of stress responses in each species. In this regard, tolerance mechanisms can be divided into two major groups: defence against osmotic stress and ion toxicity, which includes the control of ion transport and osmolyte biosynthesis and defence against oxidative stress, which includes the activation of antioxidant mechanisms.

To maintain intracellular osmotic balance, some plants accumulate toxic ions such as Na^+^ and Cl^−^ in the vacuoles [18,19]. An excessive accumulation of Na^+^ is generally accompanied by K^+^ deficiency by competition between the two cations because of their similar physicochemical properties. Thus, maintaining Na^+^/K^+^ homeostasis is crucial to develop normal metabolic processes in the cytoplasm, such as enzymatic reactions and protein synthesis [14]. In addition, different metabolites are involved in the responses to osmotic stress as osmolytes and osmoprotectants, including sugars, polyalcohols, amino acids, ammonium compounds, betaines and sulphonium compounds. Sugars are direct products of photosynthesis that play essential functions in the cell; their increase could be a response to stress or a signal for activating other cellular processes. During abiotic stress, their primary role is stress mitigation by osmoprotection, carbon storage and the scavenging of Reactive Oxygen Species (ROS). Amino acids also have some regulatory and signalling functions. In stress tolerance mechanisms, a significant role is played by the flagship compatible solute proline (Pro) [18,20,21,22].

Toxic ions absorbed by roots move into photosynthetic organs, causing harmful nutritional imbalances and the generation of oxidative stress by an increase in the production of ROS. Through the oxidation of fatty acids, amino acid residues in proteins and the DNA bases, ROS accumulation leads to membranes degradation, protein inactivation and DNA mutations, causing cellular damage, and eventually, cell death [23,24,25]. To cope with oxidative stress, plant cells activate antioxidant systems, including the activation of redox regulatory enzymes, such as superoxide dismutase (SOD), catalase (CAT), ascorbate peroxidase (APX) (and other peroxidases) or glutathione reductase (GR), and the synthesis of antioxidant metabolites, such as phenolic compounds [26].

Eggplant (*Solanum melongena* L.) is one of the most economically important crops worldwide, reaching 1.86 × 10^6^ cultivated hectares and an annual production of over 54 × 10^6^ tonnes [1]. Eggplant is moderately sensitive to salinity [27], which partially reduces growth and yield. *Solanum melongena* can be crossed with many wild relatives from the primary, secondary and tertiary gene pools, adapted to a wide range of environments [28]. Therefore, it should be possible to identify new sources of genetic variation in wild relatives adapted to saline areas. The responses of *Solanum insanum* L. to salt stress have recently been studied, and the wild genotype seems more tolerant than the cultivated eggplant, as it can stabilise its growth and photosynthetic rate under saline conditions [29]. In *S. insanum*, toxic Na^+^ and Cl^−^ ions are transported to the leaves at high external salinity, where they most likely accumulate in vacuoles, according to the ‘ion compartmentalisation hypothesis’ [30,31], whereas K^+^ concentrations are maintained. In the presence of high salt concentrations, compared with cultivated eggplant, the wild species also shows a higher accumulation of proline, an excellent marker of a stress tolerance phenotype [27].

Little is known about the inheritance of quantitative or complex traits such as stress tolerance. However, introgression can be used to introduce favourable characteristics in cultivated *S. melongena* [32,33,34] for future breeding prospects.

This study compares the response of cultivated eggplant (*S. melongena* L.), a wild relative (*S. insanum* L.) and their interspecific hybrid, under salt stress conditions in short- and long-term treatments by determining growth parameters and the levels of several biochemical stress markers—such as ions, osmolytes or antioxidant compounds—and the activity of antioxidant enzymes. This work represents the first step in assessing the possibilities of success of the introgression of salt tolerance traits from the wild relative into the cultivated species.

## 2. Results

### 2.1. Substrate Electrical Conductivity

The electrical conductivity (EC_1:5_) of the substrate in the pots was measured at the end of the short-term salt treatments of young plants. The obtained values were in concordance with the treatments applied: 4.9 dS m^−1^ on average for the control, 23.5 dS m^−1^ on average for irrigation with 200 mM of NaCl and 31.5 dS m^−1^ for irrigation with 400 mM of NaCl. No significant differences were found in these levels among the three genotypes within each treatment (Table 1).

### 2.2. Young Plant Growth Parameters

**Figure 1:**
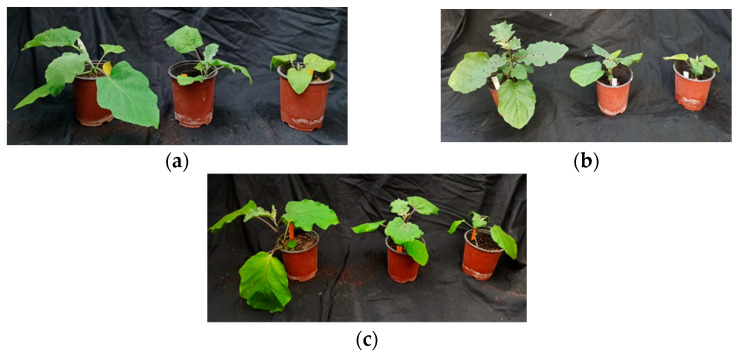
*Solanum melongena* (**a**), hybrid (**b**) and *Solanum insanum* (**c**) plants at the end of the four-week growth period, in the control, and the 200 mM NaCl and 400 mM NaCl salt treatments (from left to right).

Salt stress inhibited growth in both parents and the hybrid (Figure 1). For a better comparison among genotypes, the leaf number (LN) and the plant height (PH) are shown in the figures as their increase compared with the corresponding values at the beginning of the treatment. Root length (RL), stem diameter (SD) and leaf surface (LS) are expressed in percentages of the corresponding mean values of the control, non-stressed plants, considered as 100%.

**Figure 2:**
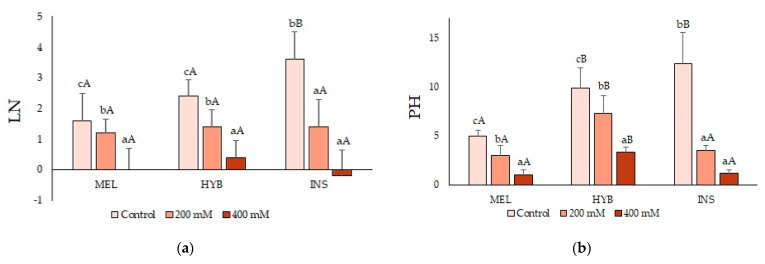
Increase in leaf number (LN) (**a**) and plant height (PH) (**b**) (mean ± SD; n = 5) in *Solanum melongena* (MEL), *Solanum insanum* (INS) and their hybrid (HYB) in comparison with the beginning of the salt treatment (control, 200 and 400 mM of NaCl, as indicated). Different lowercase letters within the same genotype indicate statistically significant differences between treatments, and different uppercase letters within the same treatment indicate statistically significant differences between genotypes (*p* < 0.05), according to the Student–Newman–Keuls multiple range test.

Under control, non-stress conditions, *S. insanum* (INS) plants apparently grew faster than the *S. melongena* (MEL) counterparts, as indicated by a larger increase in LN (Figure 2a) and PH (Figure 2b) during the four-week period, whereas values that were intermediate between those of the parents were obtained for the hybrid (HYB) plants. The three genotypes showed a significant concentration-dependent reduction in both parameters in the presence of salt (Figure 2a,b). However, the relative salt-induced growth inhibition suggested that the hybrid is somewhat more tolerant than either parent, especially at the highest salinity tested (Figure 2).

**Figure 3:**
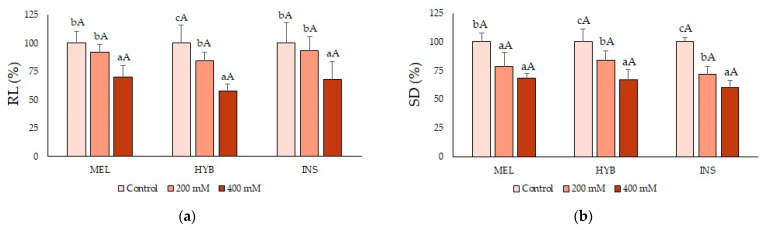

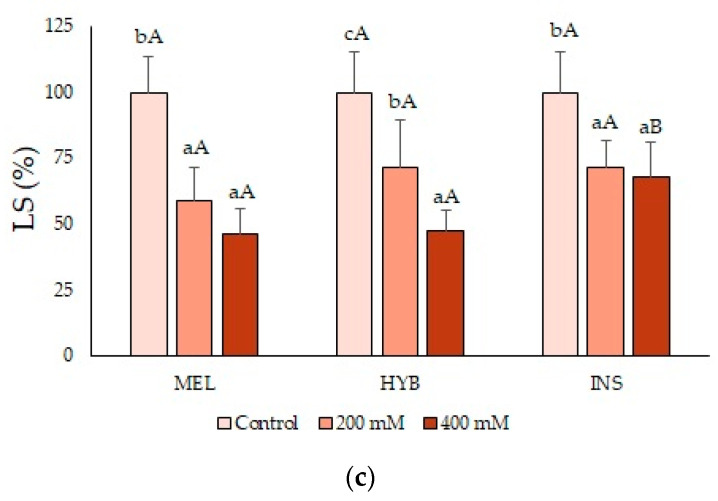
Root length (RL) (**a**), stem diameter (SD) (**b**) and leaf surface (LS) (**c**) in *Solanum melongena* (MEL), *Solanum insanum* (INS) and their hybrid (HYB) in the conditions tested (control, 200 mM and 400 mM of NaCl, as indicated). Percentages (mean ± SD; n = 5) refer to the corresponding control as 100%, with absolute values for RL (cm): [MEL = 30.4, HYB = 31.7, INS = 26.9], SD (mm): [MEL = 4.7, HYB = 5.2, INS = 5.3] and LS (cm^2^): [MEL = 187.6, HYB = 207.9, INS = 121.3]. Different lowercase letters within the same genotype indicate statistically significant differences between treatments, and different uppercase letters within the same treatment indicate statistically significant differences between genotypes (*p* < 0.05), according to the Student–Newman–Keuls multiple range test.

Similarly, the salt treatments led to a concentration-dependent reduction in RL (Figure 3a), SD (Figure 3b) and LS (Figure 3c) in all plants, compared with the corresponding non-stressed controls. These differences were always statistically significant in the presence of 400 mM of NaCl but also, in most cases, in response to the lower salinity (200 mM NaCl) treatment. Regarding these growth parameters, however, no significant differences were detected between the three tested genotypes (Figure 3).

**Figure 4:**
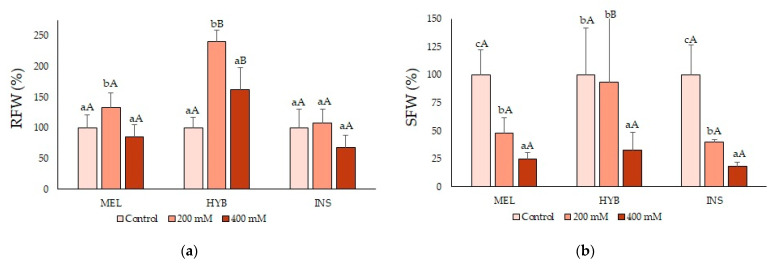

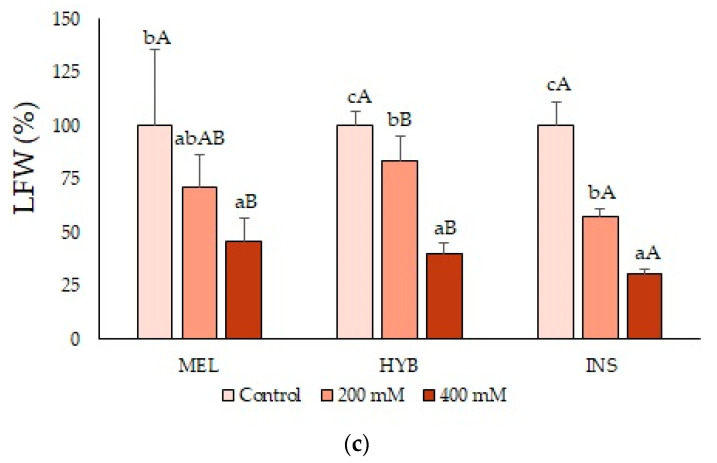
Root (RFW) (**a**), stem (SFW) (**b**) and leaf (LFW) (**c**) fresh weight in *Solanum melongena* (MEL), *Solanum insanum* (INS) and their hybrid (HYB) in the conditions tested (control 200 mM and 400 mM of NaCl, as indicated). Percentages (mean ± SD; n = 5) are referred to the control as the 100% with absolute values for RFW (g): [MEL = 13.3, HYB = 12.2, INS = 12.2], SFW (g): [MEL = 6.8, HYB = 7.7, INS = 7.4] and LFW (g): [MEL = 13, HYB = 13.1, INS = 16.2]. Different lowercase letters within the same genotype indicate statistically significant differences between treatments, and different uppercase letters within the same treatment indicate statistically significant differences between genotypes (*p* < 0.05), according to the Student–Newman–Keuls multiple range test.

Water content (WC) was homogeneous in all plants and treatments, around 80%, indicating that the salt stress did not cause dehydration in the treated plants, except for slight water stem loss for INS (Table 2). In fact, MEL and the hybrid seemed to accumulate more water in the roots and showed better maintenance of humidity than INS in both roots and leaves. Nevertheless, fresh weight (FW) was considered the most relevant growth parameter. Root fresh weight (RFW, Figure 4a) was not affected by any of the treatments in the parent species; interestingly, however, the plants of the hybrid showed a significant increase in root growth in response to the 200 mM NaCl treatment (Figure 4a). On the other hand, stem fresh weight (SFW, Figure 4b) and leaf fresh weight (LFW, Figure 4c) were significantly reduced with increasing salt concentrations. Nevertheless, the hybrid was less affected at 200 mM than any of the parents, supporting the notion of its relatively higher salt tolerance.

### 2.3. Photosynthetic Pigments

In general, no significant differences were found between treatments or genotypes in the major pigments under most experimental conditions tested (Table 3). For photosynthetic pigments, only chlorophyll *a* (ChlA) showed significantly higher values in the hybrid than in the parents in the presence of 400 mM of NaCl. The carotenoid (Caro) contents did not vary between the three genotypes, although the values calculated for INS plants were significantly lower at 400 mM of NaCl than in the control or at 200 mM of NaCl.

### 2.4. Ion Contents

**Figure 5:**
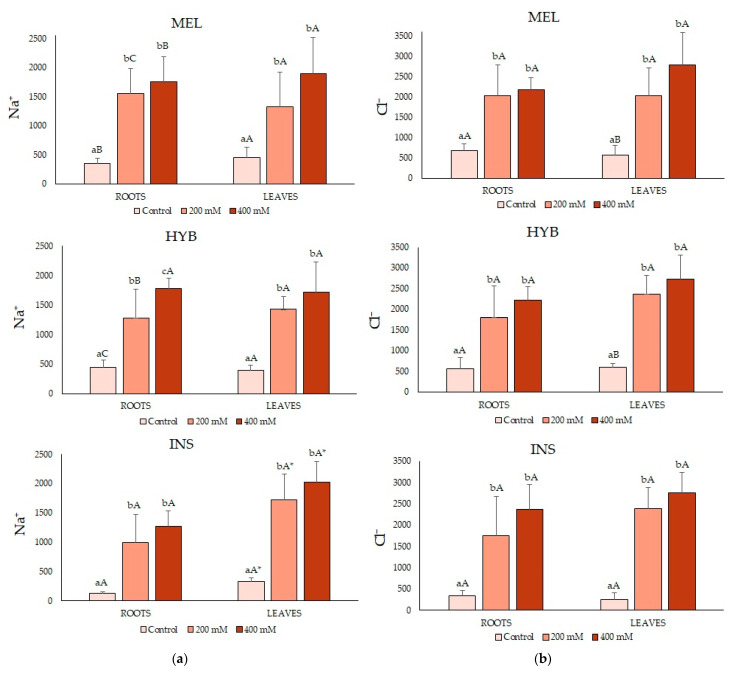

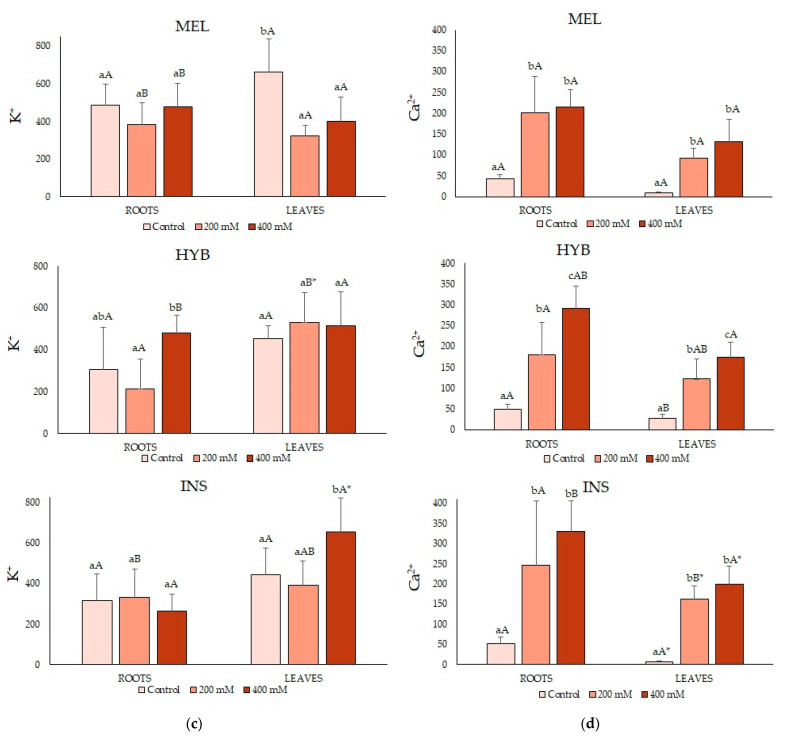
Na^+^ (**a**), Cl^−^ (**b**), K^+^ (**c**) and Ca^2+^ (**d**) content (μmol g^−1^ dry weight) in roots (left) and leaves (right) (mean ± SD; n = 5) in *Solanum melongena* (MEL), *Solanum insanum* (INS) and their hybrid (HYB) in the conditions tested (control, 200 mM and 400 mM of NaCl). Different lowercase letters within the same genotype indicate statistically significant differences between treatments, different uppercase letters within the same treatment indicate statistically significant differences between genotypes, and an asterisk indicates statistically significant differences between roots and leaves for the same treatment and genotype (*p* < 0.05), according to the Student–Newman–Keuls multiple range test.

The mean Na^+^ contents significantly increased with increasing external salinity in a concentration-dependent manner; the patterns of Na^+^ accumulation were roughly the same in roots and leaves and for the three genotypes (Figure 5a), and the absolute Na^+^ levels were also similar for each treatment, with a few exceptions. For example, the differences between 200 and 400 mM of NaCl were always non-significant, except in the roots of the hybrid, with values of 1778 and 1284 μmol g^−1^ DW, at 400 and 200 mM of NaCl, respectively. Moreover, the Na^+^ contents in INS were significantly higher in leaves than in roots for all treatments; this eggplant wild relative also showed somewhat lower Na^+^ levels in the roots compared to MEL and the hybrid (Figure 5a). The observed Cl^−^ content patterns were similar to those of Na^+^, although its absolute concentrations were somewhat higher for each treatment in both organs and the three genotypes (Figure 5b).

In the plants of all three tested genotypes, the salt treatments did not cause any significant change in K^+^ concentrations in roots compared to the corresponding non-stressed controls (Figure 5c). In leaves, however, different K^+^ accumulation patterns were observed in the two parent species upon the salt treatments: a significant salt-induced decrease of about 50% in MEL, whereas K^+^ levels significantly increased by more than 40%, in INS plants subjected to 400 mM of NaCl—although the lower salinity treatment (200 mM) had no effect. In the hybrid, leaf K^+^ concentrations neither decreased nor increased in response to the salt treatments and were maintained around 500 μmol g^−1^ DW in the control and the stressed plants (Figure 5c)

The Ca^2+^ contents followed the same pattern in both parents, in leaves and roots, significantly increasing in the presence of NaCl. In the hybrid, the measured values were intermediate between those of the parents; they showed a significant difference between 200 mM and 400 mM of NaCl. The average Ca^2+^ concentrations were higher in roots than in leaves for each salt concentration; in the case of INS, these differences (about two-fold) were statistically significant (Figure 5d).

**Figure 6:**
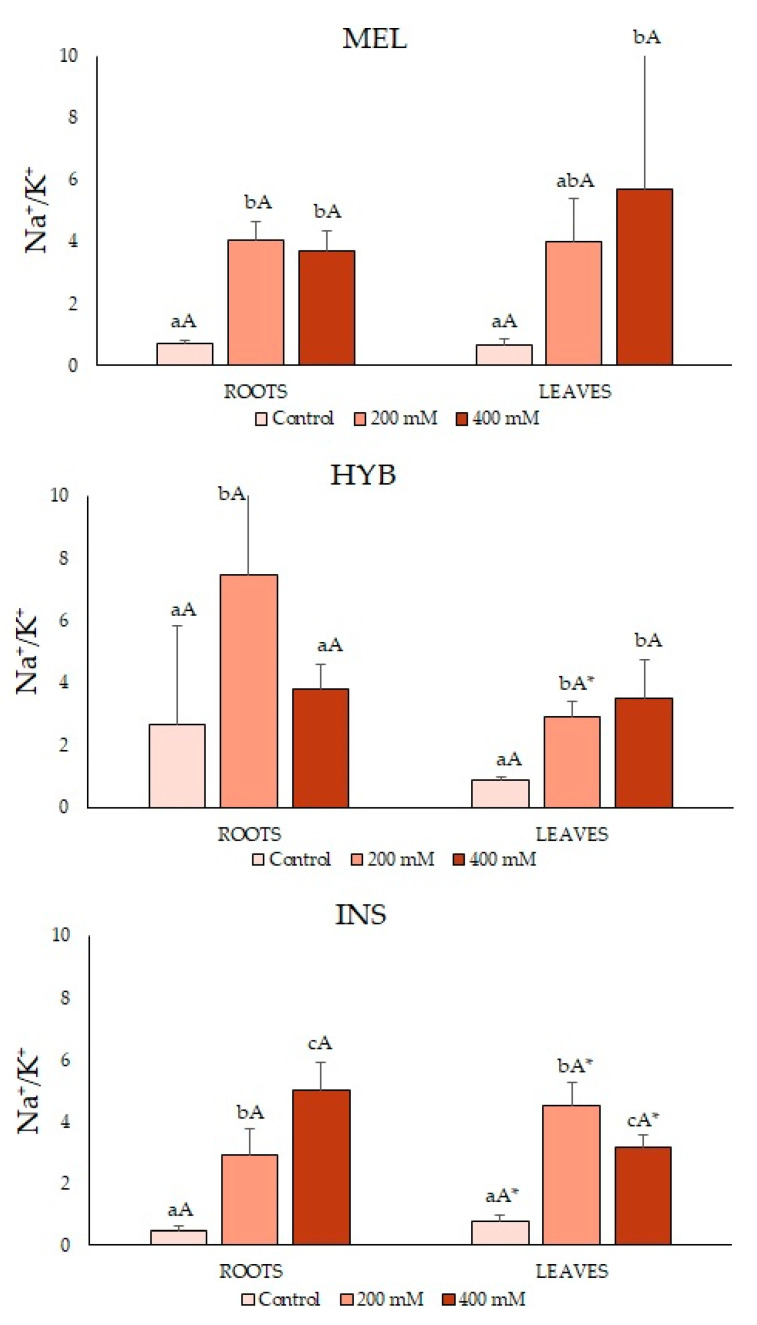
Na^+^/K^+^ relation in roots (left) and leaves (right) (mean ± SD; n = 5) in *Solanum melongena* (MEL), *Solanum insanum* (INS) and their hybrid (HYB) in the conditions tested (control, 200 mM and 400 mM of NaCl). Different lowercase letters within the same genotype indicate statistically significant differences between treatments, different uppercase letters within the same treatment indicate statistically significant differences between genotypes, and an asterisk indicates statistically significant differences between roots and leaves for the same treatment and genotype (*p* < 0.05), according to the Student–Newman–Keuls multiple range test.

The Na^+^/K^+^ ratio values were similar under the control conditions, near 1, in all samples except in hybrid roots, near 2. The ratios were higher in the salt-treated plants, with some differences between genotypes (Figure 6). In leaves, the hybrid and INS showed lower values than MEL. In roots, however, a higher average ratio was calculated for the hybrid than for both parents, at 200 mM of NaCl, although the differences were not statistically significant (Figure 6).

### 2.5. Osmolyte Quantification, MDA, H_2_O_2_ and Antioxidant Compounds

Proline (Pro) is a reliable stress marker in most species of the Solanaceae family. Leaf Pro contents increased in response to the salt treatments in the three genotypes but reached absolute values about 25-fold higher in INS and the hybrid than in MEL (Table 4). In the non-stressed controls, the hybrid tended to accumulate more Pro than both parents. Under salt stress conditions, both the hybrid and INS accumulated more Pro than MEL.

The total soluble sugars (TSS) contents did not vary significantly between treatments or genotypes, with values around 200 mg equiv. glu g^−1^ DW in all cases (Table 4). Similarly, the levels of oxidative stress biomarkers (MDA, H_2_O_2_) and total phenolic compounds (TPC, representative antioxidant metabolites), in general, did not significantly increase in response to the salt treatments, except for H_2_O_2_ in INS plants watered with 400 mM of NaCl (Table 4). Total flavonoids (TF) levels showed significant differences between genotypes in the control and 200 mM NaCl treatments, with the values of the hybrid closer to the wild parent species than the cultivated eggplant; however, no differences were detected between treatments for each genotype (Table 4).

### 2.6. Antioxidant Enzymes

The salt treatments did not increase the specific activity of relevant antioxidant enzymes, such as superoxide dismutase (SOD), aspartate peroxidase (APx) or glutathione reductase (GR) in any of the studied genotypes (Table 5), as should be expected, since no salt-induced oxidative stress was observed. Under the assay conditions, no catalase (CAT) activity could be detected (data not shown). The enzymes activities showed great variability within the same genotype and treatment, as shown by the relatively high SD values.

### 2.7. Adult Plant Performance

To complement the previous data, adult plants at the pre-flowering stage were subjected to longer (eight weeks) treatments with lower (80 mM of NaCl) salt concentrations. After the treatment, growth parameters, root and leaf ion contents, and leaf Pro levels were determined in the plants of MEL, INS and their hybrid.

**Figure 7:**
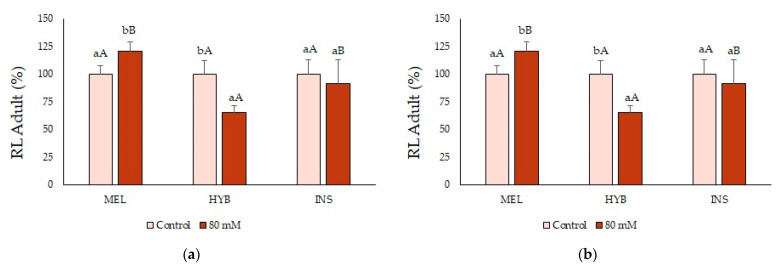
Root length (RL) (**a**) and leaf surface (LS) (**b**) (mean ± SD; n = 5) in adult plants of *Solanum melongena* (MEL), *Solanum insanum* (INS) and their hybrid (HYB) in control conditions and under long-term (eight weeks) 80 mM NaCl irrigation. Values are shown as percentages of the corresponding controls of each genotype, taken as 100%, with absolute values for RL (cm): [MEL = 65.2, HYB = 68.6, INS = 71.6] and LS (cm^2^): [MEL = 141, HYB = 172.4, INS = 140.8]. Different lowercase letters within the same genotype indicate statistically significant differences between treatments, and different uppercase letters within the same treatment indicate statistically significant differences between genotypes (*p* < 0.05), according to the Student–Newman–Keuls multiple range test.

Regarding root length (RL), the salt treatment caused a small but significant increase in MEL plants, whereas it did not affect INS; on the other hand, RL was significantly reduced in HYB plants in the presence of 80 mM of NaCl (Figure 7a). The mean leaf surface (LS) values saw a similar decrease in the three genotypes, although to a somewhat lesser extent in MEL plants (Figure 7b).

**Figure 8:**
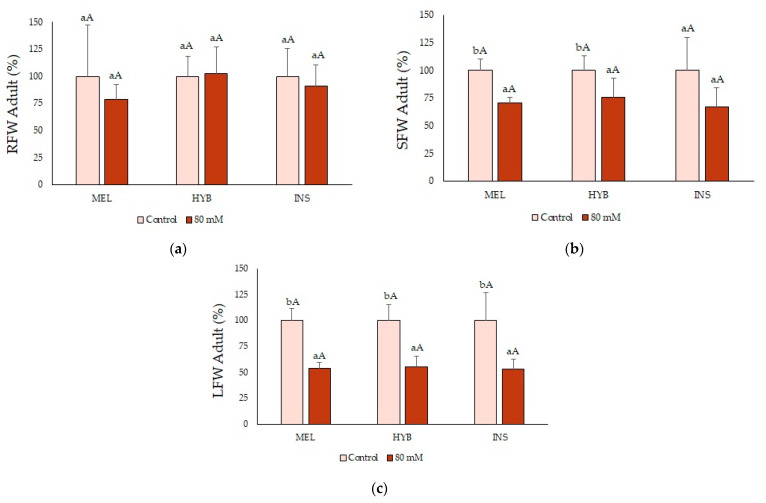
Root (RFW) (**a**), stem (SFW) (**b**) and leaf (LFW) (**c**) fresh weight (mean ± SD; n = 5) in adult plants of *Solanum melongena* (MEL), *Solanum insanum* (INS) and their hybrid (HYB) in control conditions and under long term (eight weeks) 80 mM NaCl irrigation. Values are shown as percentages of the corresponding controls of each genotype, taken as 100%, with absolute values for RFW (g): [MEL = 129.9, HYB = 177.4, INS = 111.2], SFW (g): [MEL = 120.1, HYB = 185.3, INS = 131.2] and LFW (g): [MEL = 130.2, HYB = 195.1, INS = 174]. Different lowercase letters within the same genotype indicate statistically significant differences between treatments, and different uppercase letters within the same treatment indicate statistically significant differences between genotypes (*p* < 0.05), according to the Student–Newman–Keuls multiple range test.

No effect of the salt treatment on biomass accumulation was observed at the root level (Figure 8a). However, salt stress significantly reduced the growth of the aerial part of the plants to a similar degree for MEL, INS and their hybrid, as shown by the decrease in SFW and LFW (Figure 8b,c).

**Figure 9:**
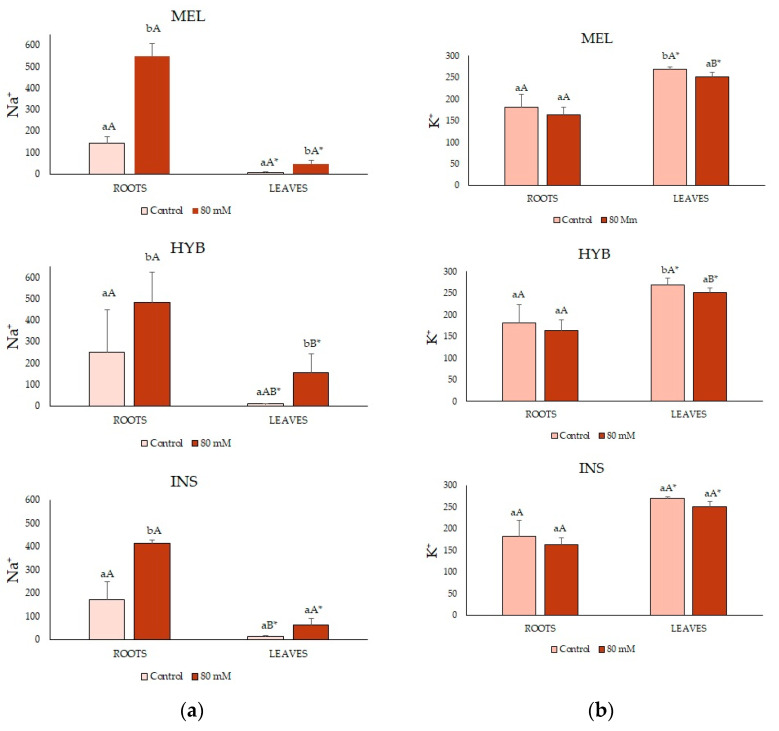

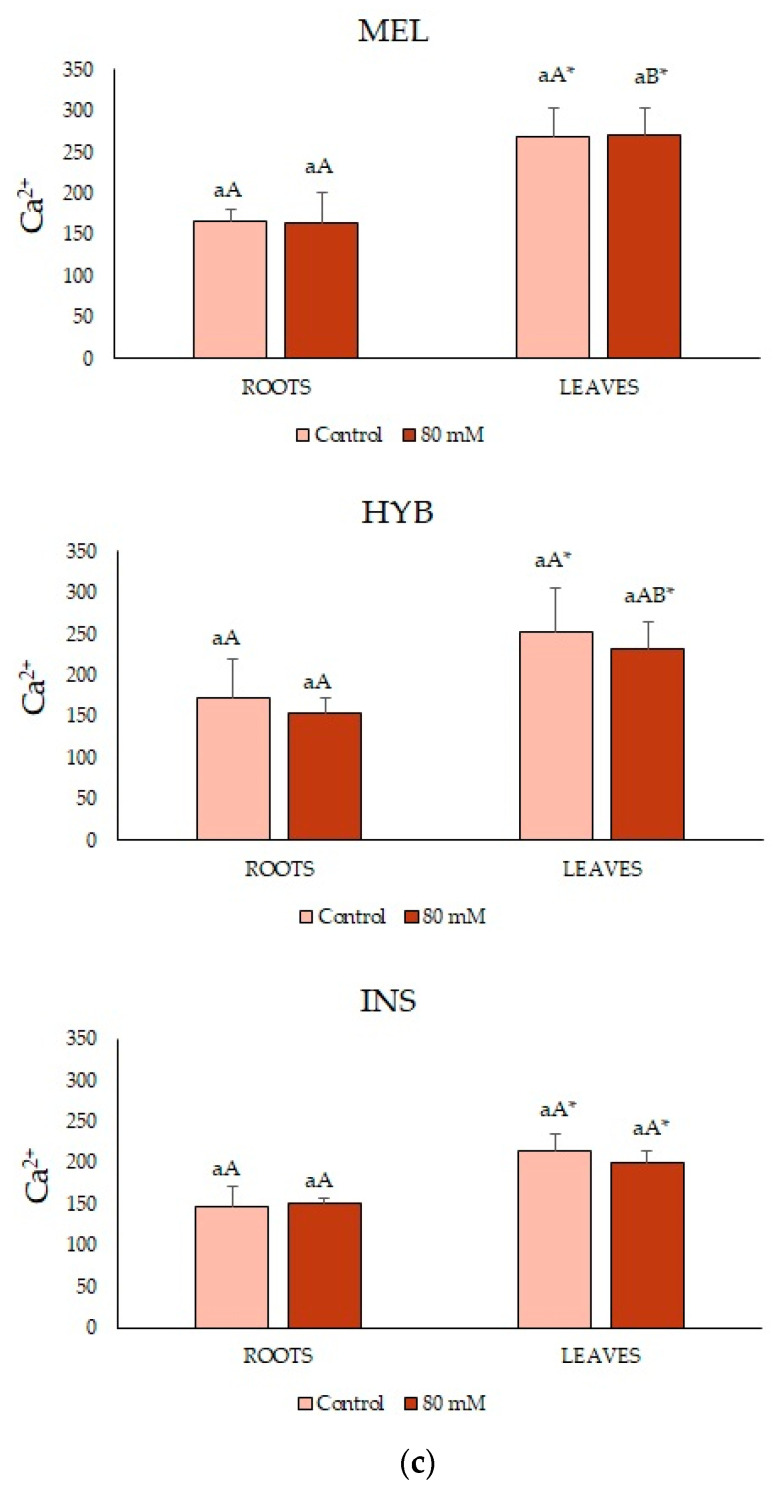
Na^+^ (**a**), K^+^ (**b**), and Ca^2+^ (**c**) content (μmol g^−1^ dry weight) in roots (left) and leaves (right) (mean ± SD; n = 5) in *Solanum melongena* (MEL), *Solanum insanum* (INS) and their hybrid (HYB) in the conditions tested (control, 80 mM of NaCl). Different lowercase letters within the same genotype indicate statistically significant differences between treatments, different uppercase letters within the same treatment indicate statistically significant differences between genotypes, and an asterisk indicates statistically significant differences between roots and leaves for the same treatment and genotype (*p* < 0.05), according to the Student–Newman–Keuls multiple range test.

The adult plants showed significant differences between leaf and root ion content for all analysed ions and the three tested genotypes. Thus, Na^+^ concentrations were much higher in roots than in leaves; K^+^ and Ca^2+^, on the contrary, accumulated to significantly higher levels in leaves than in roots, although the differences between organs were relatively small compared to those observed for Na^+^ (Figure 9).

Regarding the effect of the salt treatment on ion levels, Na^+^ concentrations increased after eight weeks in the presence of 80 mM of NaCl, as expected; the differences with the controls were statistically significant in all samples, except in the leaves of INS plants (Figure 9a). Interestingly, the leaf Na^+^ contents were significantly higher in the hybrid than in its two parents (Figure 9a). Contrary to Na^+^, mean K^+^ (Figure 9b) and Ca^2+^ (Figure 9c) concentrations varied little in response to the salt treatment, neither in the roots nor in the leaves of the plants of the three genotypes. Indeed, a slight NaCl-induced reduction in leaf K^+^ contents in MEL and HYB plants was the only significant difference detected (Figure 9b). It should also be pointed out that the mean K^+^ and Ca^2+^ leaf contents were higher than those of Na^+^ for the three analysed genotypes (Figure 9).

**Figure 10:**
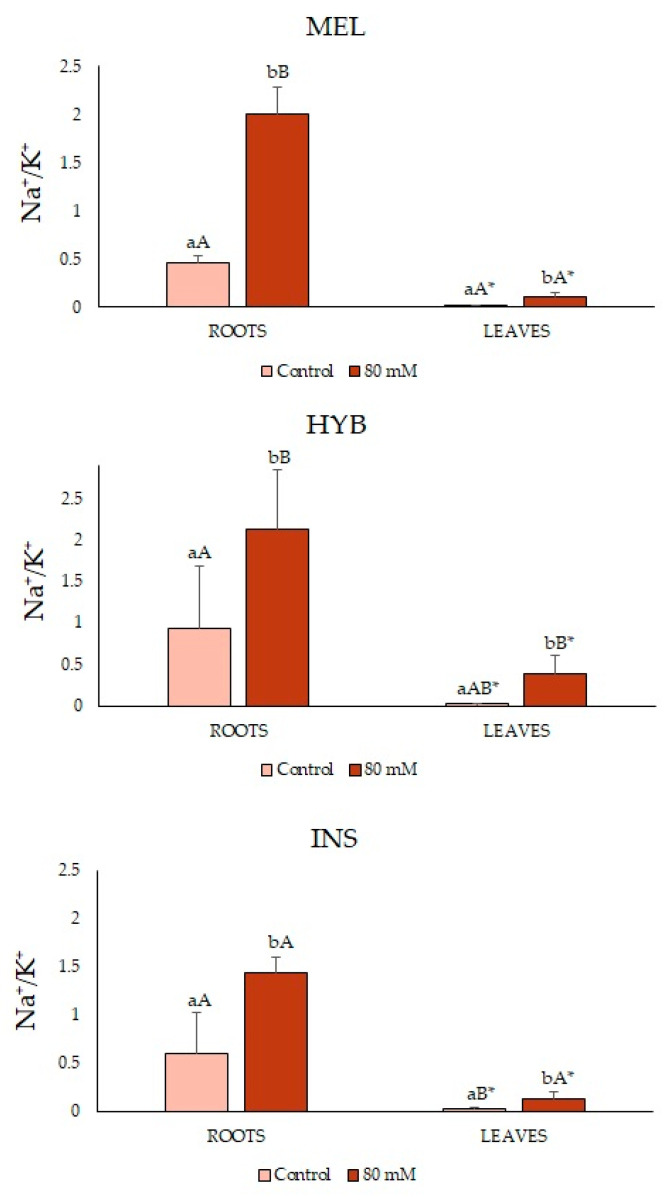
Na^+^/K^+^ ratio in roots (left) and leaves (right) (mean ± SD; n = 5) in *Solanum melongena* (MEL), *Solanum insanum* (INS) and their hybrid (HYB) in the conditions tested (control, 80 mM of NaCl). Different lowercase letters within the same genotype indicate statistically significant differences between treatments, different uppercase letters within the same treatment indicate statistically significant differences between genotypes, and an asterisk indicates statistically significant differences between roots and leaves for the same treatment and genotype (*p* < 0.05), according to the Student–Newman–Keuls multiple range test.

The Na^+^/K^+^ ratios remained low, below 1.0, under the control conditions for the three genotypes tested, especially in leaves, which showed much lower values than roots. These ratios significantly increased, in all cases, in response to the NaCl treatment (Figure 10).

The leaf Pro contents increased significantly in response to the salt treatment in MEL and HYB plants, about ten- and five-fold, respectively, with respect to the corresponding controls. The mean Pro content also increased in salt-treated INS plants, but the difference with the control was not statistically significant (Table 6). Therefore, in the hybrid, absolute Pro levels under control and salt stress conditions, and their relative increase, were intermediate between those of the parent species.

## 3. Discussion

Recent studies have revealed that, even though *Solanum melongena* has been described as moderately susceptible—or tolerant—to salt stress, the response largely depends on the genotype [35,36,37,38]. The effects of salt stress on plant development are well established [3,16], although specific response mechanisms depend on the genotype and the experimental conditions tested [29,39]. The present work compared the behaviour of an eggplant cultivar (MEL), the wild relative *Solanum insanum* (INS) and their hybrid (HYB), with the aim of establishing possible differences in salt tolerance between the tested genotypes and the inheritance of those tolerance mechanisms. Our general assumption was that both parents, closely related genetically, would not differ in the type of responses to salt stress but could differ in the magnitude or efficiency of those responses.

High salt concentrations cause growth inhibition in plants [35,36,40], which was also observed in the present work for the three studied genotypes, especially at 400 mM NaCl. Some quantitative differences between both parents were detected in our experiments; for example, the wild relative seemed to grow more rapidly than the cultivated eggplant in the absence of salt, as shown by the larger increase in leaf number and plant height. However, considering most measured growth variables, MEL and INS plants did not differ substantially in terms of their relative growth inhibition under salt stress, indicating that the degree of salt tolerance was similar for the tested *S. melongena* cultivar and *S. insanum.* However, in a previous study, another *S. melongena* cultivar was shown to be less salt-tolerant than the wild relative at high salinities [29], confirming the genotype dependence of this trait in eggplant. On the other hand, our results pointed to a higher tolerance of the hybrid than either parent: a smaller relative reduction in the leaf surface and plant height and, most importantly, a relatively higher biomass accumulation (FW of stems and leaves) in the presence of 200 mM of NaCl. Moreover, while root FW did not change in response to the salt treatments in MEL and INS plants, it significantly increased in hybrid plants treated with 200 mM of NaCl. Since the primary root length actually decreased, the intermediate salt treatment most likely induced the growth of secondary roots to enhance water uptake from the soil, as has been observed in some other species [41,42,43,44], suggesting a specific adaptation to salt stress in the hybrid. The higher efficiency of the defence mechanisms against salt stress observed in the hybrid may relate to the well-known phenomenon of heterosis that hybrids show in comparison to their parents, leading to improving important agronomical quantitative traits, such as growth vigour, seed production, yield or even stress survival [45,46]. Heterosis has been observed and studied in many different plant species, including, for example, maize, rice or tomato [47,48,49], as well as in eggplant [50]. This hybrid vigour would be of great interest in future eggplant breeding prospects, but the genetic basis of heterosis is still under study.

Salinity is generally associated with the degradation of photosynthetic pigments, which is more pronounced in less tolerant species, reducing photosynthesis activity in the plant. Consequently, chlorophylls *a* and *b* (ChlA and ChlB), and carotenoid (Caro) contents, might be used as stress biomarkers [51,52]. In the present work, however, we did not observe a significant salt-induced reduction in ChlA, ChlB or Caro in any of the genotypes, thus, supporting the moderate salt tolerance of eggplant and its wild relative. Interestingly, the ChlA contents were higher in the hybrid than in both parents in the presence of 400 mM of NaCl, which was probably also due to an heterotic effect.

Na^+^ and Cl^−^ contents accumulated in leaves and roots when salinity increased, with similar patterns for *S. melongena, S. insanum* and the hybrid, as previously reported in different eggplant cultivars [53,54]. However, we did not observe significant differences between roots and leaves, except for *S. insanum*, with a more significant Na^+^ accumulation in leaves. This result supports the idea that in *S. insanum*, Na^+^ is transported from roots to leaves and is sequestered in leaf vacuoles with a somewhat higher efficiency than in the cultivated eggplant.

An increase in Na^+^ contents in response to increasing salinity would generally promote a decrease in K^+^ concentration, as both cations compete for the same transport proteins of the membrane [36,53,54]. For this reason, the Na^+^/K^+^ ratio has been reported as a possible indicator of salt tolerance in plants [14,55]. Some studies revealed a limited Na^+^ accumulation in eggplant leaves to maintain lower Na^+^/K^+^ ratios, reducing Na^+^ uptake or compartmentalising it into root vacuoles [56,57]. In this study, we observed that the plants of the three genotypes maintained root K^+^ levels under salt stress, which likely contributes to salt tolerance in eggplant, its wild relative and their hybrid. Changes in leaf K^+^ concentrations were also not detected in salt-treated HYB plants, which in this respect showed an intermediate behaviour between its parent species. The Na^+^ contents measurements indicated that ion transport in response to salt differs in both parental species. In INS, Na^+^ levels were higher in leaves, whereas Na^+^ predominantly accumulated in roots in MEL and the HYB, which in this respect was more similar to the cultivated eggplant than the wild relative.

The Ca^2+^ contents increased in the presence of salt, with higher accumulation in roots than in leaves, supporting the notion that Ca^2+^ is beneficial for maintaining Na^+^ and K^+^ homeostasis, at least partly via the SOS pathway [58]. Moreover, due to its critical regulatory and signalling roles in plant growth and development [59], increasing Ca^2+^ in the leaves of salt-stressed plants is important to maintain essential metabolic and cellular processes.

Proline (Pro) is a reliable marker of abiotic stress in many plant species, increasing its concentration in stressed plants in relation to its background levels in non-stressed controls [15,60]. In addition to its role in osmotic adjustment, Pro is an osmoprotectant because of its function as a low-molecular-weight chaperon, maintaining protein structure and membrane integrity, as well as ROS detoxification under stress conditions [61]. Several reports have shown a positive correlation between Pro accumulation and stress tolerance when comparing related taxa, suggesting the direct participation of Pro in the mechanisms of tolerance [20,62,63]. In other cases, however, Pro contents simply indicate the relative degree of stress affecting the plants, accumulating at higher levels in the more-stressed, less-tolerant genotypes, indicating that Pro is not directly involved in relevant tolerance mechanisms [37,38,64]. Our results showed higher Pro contents in the control plants of the hybrid than in *S. melongena* or *S. insanum*, suggesting a constitutive mechanism of stress response. The salt treatments led to significant, concentration-dependent increases in Pro accumulation in all three genotypes; however, the absolute concentrations reached were much higher, around 25-fold, in INS and HYB than in MEL. Therefore, regarding Pro biosynthesis and accumulation in response to salt stress, the hybrid was much closer to the wild relative than the cultivated eggplant.

Soluble sugars (TSS) play a role in osmoregulation under stress environments in some plant species, amongst other multiple functions unrelated to specific responses to stress [65,66]. This report does not show any significant variation in TSS contents in response to salt stress, indicating that sugar accumulation is not a relevant mechanism of salt tolerance in eggplant.

Malondialdehyde (MDA), a product of lipid peroxidation, is an excellent marker of oxidative stress generated as a secondary effect of high salinity and other abiotic stresses due to the increase in cellular ROS levels [67]. Therefore, in general, it should be expected that MDA contents increase in plants in the presence of salt, as should the levels of antioxidant compounds and enzyme activities, in order to counteract oxidative stress [68,69,70]. In this study, however, MDA contents did not vary significantly in response to the salt treatments. Similarly, no salt-induced increase in H_2_O_2_ contents was detected, in agreement with the maintenance of low ROS levels, but for a slight increase in INS at 400 mM of NaCl. These data suggest that salt stress responses based on the control of ion transport and Pro accumulation are efficient enough to avoid the generation of oxidative stress in salt-treated MEL, INS or HYB plants under our experimental conditions. In the absence of oxidative stress, the activation of antioxidant enzymes or the synthesis of antioxidant metabolites, such as phenolic compounds, was not detected, as should be expected.

The responses of adult plants to lower salt concentrations during longer treatment were qualitatively similar, in most cases, to those described above for younger plants, albeit generally weaker. This behaviour could be explained by the increase in stress tolerance with plant age, which has been observed in many plant species [71,72,73]. The salt treatment also caused an inhibition of growth in adult plants, mainly reflected in a reduction in the FW of the aerial part, stem and leaves, but without significant differences between the three genotypes.

The Na^+^ contents significantly increased in salt-treated plants with respect to the non-treated controls, in roots and leaves. However, contrary to what was observed in younger plants, Na^+^ concentrations were much lower in leaves than in roots. It seems that adult plants can efficiently block the transport of the toxic ion to the aerial part of the plants. On the other hand, K^+^ and Ca^2+^ concentrations were slightly (but significantly) higher in leaves than in roots and did not vary in response to the salt treatment or between genotypes. Compared to young plants, where Ca^2+^ is accumulated primarily in roots, Ca^2+^ concentrations were slightly but significantly higher in leaves in adult plants. Moreover, Na^+^/K^+^ ratios were significantly lower in adult hybrid roots compared to young hybrid roots. The relatively low accumulation in the leaves of Na^+^ ions, and the higher contents of K^+^ and Ca^2+^, partly counteracting the deleterious effects of salt stress, most likely contribute to the higher tolerance of adult plants.

Finally, Pro contents increased in the plants treated with 80 mM of NaCl, although the relative increase over control levels and the maximum values reached were lower than in young plants. In this case, however, the lowest Pro concentrations were measured in INS plants and the highest in MEL, with the hybrid showing intermediate values. Since toxic Na^+^ accumulated in leaves at much lower levels than in young plants, it seems logical to assume that lower Pro concentrations are also required for osmotic adjustment.

## 4. Materials and Methods

### 4.1. Plant Material, Seed Germination and Plant Growth

Seeds of *Solanum melongena* (MEL), *Solanum insanum* (INS) and their hybrid *S. insanum × S. melongena* (HYB), originally obtained from Sri Lanka and maintained in the COMAV eggplant collection (Universitat Politècnica de València, Spain) [28], were sown following the protocol of Ranil et al. [74]. The seeds were immersed for 24 h in distilled water, followed by 24 h in a solution of 1.4 mM of gibberellic acid (GA). After the treatment, the seeds were placed in standard Petri dishes (90 mm in diameter) with a sterile cotton layer covered with filter paper and wetted with 10 mM of potassium nitrate (KNO_3_). The seeds were incubated at 4 °C covered with aluminium foil for seven days, followed by 24 h at 37 °C, before placing them in the germination chamber (25 °C, 78% humidity and a photoperiod of 16/8 h with a mean light intensity of 1000 lux).

Plants with four leaves were transplanted in the greenhouse into pots of 14.5 cm in diameter, 12.5 cm in height (1.3 L Series CD Alto Thermoformed Pots) with commercial soil (Neuhaus Huminsubstrat S), and a 1 cm layer of vermiculite; the pots were placed in plastic trays distributed in groups of ten. One week later, treatments were started.

### 4.2. Salt Treatments

Five plants of each genotype were watered with solutions containing either 200 or 400 mM of NaCl, or with water for the controls, every three days for four weeks (n = 5). At the beginning of the treatments, the number of leaves (NL) and plant height (PH) (cm) were measured in all plants. At the end of the experiment, the same parameters were measured, plus the following: stem diameter (SD) (mm), length of the primary root (LR) (cm), leaf surface (LS) (cm^2^), and the electrical conductivity of the substrate (EC) (dS m^−1^). Once these parameters were determined, the plants were taken into the laboratory to measure the leaf fresh weight (g) (LFW), shoot fresh weight (g) (SFW) and root fresh weight (g) (RFW). Part of the fresh leaf material was deep-frozen in liquid N_2_ to perform some of the biochemical assays. Part of the fresh material was weighed and dried for three days at 65 °C and then weighed again. The water content (WC) percentage was calculated using Equation (1) [22].

WC (%) = [(FW − DW)/FW] × 100(1)

### 4.3. Substrate Electrical Conductivity

The EC of the substrate was measured at the end of the treatments using a 1:5 soil:water suspension prepared in deionised water and stirred for two h at 600 rpm and 4 °C. EC_1:5_ (dS m^−1^) was measured with a Crison Conductivity-meter 522 (Crison Instruments SA, Barcelona, Spain).

### 4.4. Photosynthetic Pigments

Total carotenoids (Caro), chlorophyll *a* (ChlA) and chlorophyll *b* (ChlB) were measured following the procedure of Lichtenthaler and Wellburn [75], as modified in [76]. Pigments were extracted from 0.05 g of fresh leaf material in 10 mL of 80% ice-cold acetone. After mixing overnight and centrifuging for 10 min at 13,300× *g*, the supernatant was collected, and its absorbance was measured at 663, 646 and 470 nm. Pigment concentrations were calculated following the equations given by Lihtenthaler and Wellburn [75], and pigment contents were expressed in mg g^−1^ DW.

### 4.5. Ion Content Measurement

The ion contents were determined in samples of 0.05 g of dry leaf, stem and root material after extraction in 15 mL of H_2_O, according to the original protocol of Weimberg [77], with minor modifications [78]. The samples were heated at 95 °C for one hour in a water bath, mixed overnight in a rocker shaker, and centrifuged for 10 min at 13,300× *g*. Cations (Na^+^, K^+^ and bioavailable Ca^2+^) were quantified with a PFP7 flame photometer (Jenway Inc., Burlington, VT, USA), and Cl^-^ was measured using a chloride analyser Corning 926 (Sherwood Scientific Ltd., Cambridge, UK).

### 4.6. Osmolyte Quantification

Proline (Pro) was extracted from 0.05 g of fresh leaf material with two mL of 3% (*w*/*v*) sulphosalicylic acid and quantified according to the ninhydrin method [79], as described [29]. The extract mixed with acid ninhydrin was heated at 95 °C in a water bath for one hour, cooled on ice and extracted with toluene; the absorbance of the organic phase was measured at 520 nm. Samples with known Pro amounts were assayed in parallel to obtain a standard curve. Pro concentrations were expressed as μmol g^−1^ DW.

The total soluble sugars (TSS) were quantified using 0.05 g of fresh leaf material. The samples were extracted with 3 mL of 80% (*v*/*v*) methanol and mixed overnight. The extracts were centrifuged for 10 min at 13,300× *g*, concentrated sulphuric acid and 5% phenol were added to the supernatant, and the absorbance was measured at 490 nm. The TSS contents were expressed as equivalents of glucose, used as the standard (mg eq. gluc g^−1^ DW) [80,81].

### 4.7. MDA, H_2_O_2_ and Antioxidant Compounds

The malondialdehyde (MDA) contents were determined following the method described by Hodges et al. [82], with some modifications [78]. Leaf methanol extracts (80% *v*/*v*) were supplemented with 0.5% thiobarbituric acid (TBA) in 20% trichloroacetic acid (TCA), or with 20% TCA without TBA for the controls, and incubated for 15 min at 95 °C in a water bath. The reaction was stopped on ice, and the absorbance was measured at 440, 600 and 532 nm. The MDA concentrations were calculated using the equations described in [83].

The hydrogen peroxide (H_2_O_2_) contents were quantified using the method described by Loreto and Velikova [84]. An amount of 0.05 g of fresh leaf material was extracted with a 0.1% (*w*/*v*) TCA solution and centrifuged for 10 min at 13,300× *g*. The supernatant was mixed with one volume of potassium phosphate buffer (10 mM, pH 7.0) and two volumes of 1 M potassium iodide. The absorbance was measured at 390 nm, and H_2_O_2_ concentrations were calculated against a standard calibration curve and expressed as μmol g^−1^ DW.

Total phenolic compounds (TPC) and total flavonoids (TF) were determined in the same methanol extracts used for the TSS measurement. TPC were quantified by reaction with Folin–Ciocalteu reagent [85]. The absorbance was measured at 765 nm, and the results were expressed as equivalents of gallic acid, used as the standard (mg equiv. acid gallic g^−1^ DW). TF were measured following the method described by Zhishen et al. [86], based on the nitration of phenolic rings containing a catechol group, followed by reaction with AlCl_3_ at basic pH. The absorbance was measured at 510 nm, and TF contents were expressed in equivalents of the standard catechin (mg equiv. cathechin g^−1^ DW).

### 4.8. Antioxidant Enzymes Activities

Crude protein extracts were prepared from leaf material, as previously described [20], and stored at −75 °C. The protein concentration was determined according to Bradford [87,88], using the Bio-Rad reagent and bovine serum albumin (BSA) as the standard. The specific activities in the protein extracts of the four selected antioxidant enzymes were determined by spectrophotometric assays.

Superoxide dismutase (SOD) activity was determined by monitoring, at 560 nm, the inhibition of nitroblue tetrazolium (NBT) photoreduction in reaction mixtures containing riboflavin as the source of superoxide radicals [89,90]. One SOD unit is defined as the amount of enzyme causing 50% inhibition of the NBT photoreduction.

Catalase (CAT) activity was measured following the consumption of H_2_O_2_ added to the extracts by the decrease in absorbance at 240 nm [90,91]. One CAT unit is defined as the amount of enzyme necessary to decompose one mmol of H_2_O_2_ per min at room temperature.

Ascorbate peroxidase (APx) activity was determined following the decrease in absorbance at 290 nm, caused by oxidation of the ascorbate present in the plant extract [92,93]. One APx unit is defined as the amount of enzyme catalysing the consumption of one mmol of ascorbate per min at room temperature.

Glutathione reductase (GR) activity was quantified by the decrease in absorbance at 340 nm, the oxidation of nicotinamide adenine dinucleotide phosphate (NADPH), and the cofactor in the GR-catalysed reduction in oxidised glutathione (GSSG) [94,95]. One GR unit is defined as the amount of enzyme required to oxidise one mmol of NADPH per min at room temperature.

### 4.9. Adult Plant Evaluation

Before the salt treatments, ten plants per genotype were transplanted into pots of 33 cm diameter and 28.5 cm height with coconut fibre. A controlled-release fertiliser, CoteN Mix, with NPK = 20−5−10, was added to each pot. Fertilisation was repeated after four weeks. Five plants were maintained until the pre-flowering stage when a long-term salt treatment started, by watering with a solution containing 80 mM of NaCl (n = 5). Automatised irrigation was set to water each plant with 1.65 litres daily. Once a week, the plants were irrigated with a control solution without salt to avoid salt accumulation in the coconut fibre and the pot.

After eight weeks, growth parameters (RL, LS, LFW, SFW and RFW) were determined, as well as the DW and WC of leaves, stem and roots, as described above for young plants.

Considering the results obtained for young plants, only ions and Pro were quantified in adult plants. Na^+^, K^+^ and Ca^2+^ were measured using 2 g of dry leaf or root material in an Agilent ICP-EOS 710 photometer (Agilent Technologies, Santa Clara, CA, USA). Pro contents were determined following the protocol described in 4.7.

### 4.10. Statistical Analysis

Data were analysed using the software Statgraphics Centurion v.XVII (Statpoint Technologies, Warrenton, VA, USA). A factorial analysis of variance was performed for all parameters analysed in the plants, considering two factors of variability—treatments and genotypes—and their interaction. An analysis of variance was carried out separately for each species. Differences between the treatments were evaluated by the Student–Newman–Keuls test.

## 5. Conclusions

Our results confirm the relatively high tolerance of eggplant to moderate salinity compared to most common crops, primarily based on the control of ion transport and Pro accumulation. These mechanisms seem efficient enough to avoid the generation of oxidative stress and, therefore, the activation of antioxidant systems as an additional defence mechanism.

The hybrid *S. melongena x S. insanum* showed a heterotic effect on plant growth, being more salt-tolerant than either parent. It appeared closer to the wild species in some crucial responses, such as the NaCl-dependent Pro accumulation in young plants. Wild relatives have been exploited to introgress novel genes that increase the fitness of the cultivated species. The results presented here suggest that *S. insanum* and the hybrid can be used as new sources of variation for breeding programmes, in order to obtain eggplant varieties that are better adapted to salt stress and, consequently, more sustainable.

## Figures and Tables

**Table 1:** Electrical conductivity values (EC_1:5_ mean ± SD; n = 5) in the pot substrates of *Solanum melongena* (MEL)*, Solanum insanum* (INS) and their hybrid (HYB), when applying the salt treatments. Different lowercase letters within the same row indicate statistically significant differences between treatments, and different uppercase letters within the same column indicate statistically significant differences between genotypes (*p* < 0.05), according to the Student–Newman–Keuls multiple range test.

		Treatment (NaCl Concentrations)		
		Control	200 mM	400 mM
EC_1:5_	MEL	5.2 ± 1.6 ^aA^	21.8 ± 3.2 ^bA^	32.2 ± 4.1 ^cA^
	HYB	5.2 ± 2.001 ^aA^	26.4 ± 3.2 ^bA^	31 ± 5.9 ^bA^
	INS	4.4 ± 1.9 ^aA^	22.1 ± 2.9 ^bA^	31.8 ± 3.9 ^cA^

**Table 2:** Root (RWC) (**a**), stem (SWC) (**b**) and leaf (LWC) (**c**) water content (%) (mean ± SD; n = 5) in *Solanum melongena* (MEL), *Solanum insanum* (INS) and their hybrid (HYB) in the conditions tested (control 200 mM and 400 mM of NaCl, as indicated). Different lowercase letters within the same genotype indicate statistically significant differences between treatments, and different uppercase letters within the same treatment indicate statistically significant differences between genotypes (*p* < 0.05), according to the Student–Newman–Keuls multiple range test.

		Treatment (NaCl Concentrations)		
		Control	200 mM	400 mM
RWC	MEL	77.6 ± 1.4 ^aA^	84.2 ± 1.2 ^bB^	83.5 ± 0.9 ^bB^
	HYB	75.4 ± 1.6 ^aA^	83.1 ± 0.5 ^bB^	83.6 ± 0.4 ^bB^
	INS	77.5 ± 1.8 ^aA^	78.3 ± 0.4 ^aA^	78.2 ± 0.3 ^aA^
SWC	MEL	90.1 ± 4.5 ^bA^	84.3 ± 1.7 ^aA^	83.5 ± 0.9 ^aA^
	HYB	79.9 ± 11.3 ^aA^	77.3 ± 26.2 ^aA^	75.1 ± 18.9 ^aA^
	INS	78.7 ± 3.02 ^bA^	76.2 ± 1.8 ^bA^	71.1 ± 0.9 ^aA^
LWC	MEL	81.8 ± 5.1 ^aA^	86.1 ± 2.4 ^aA^	85.6 ± 1.4 ^aB^
	HYB	87.9 ± 6.3 ^aA^	85.9 ± 0.9 ^aA^	83.6 ± 0.4 ^aB^
	INS	82.6 ± 3.2 ^aA^	83.8 ± 1.3 ^aA^	81.2 ± 1.5 ^aA^

**Table 3:** Chlorophyll *a* (ChlA) (mg g^−1^ dry weight), chlorophyll *b* (ChlB) (mg g^−1^ dry weight) and carotenoids (Caro) (mg g^−1^ dry weight) values (mean ± SD; n = 5) in *Solanum melongena* (MEL), *Solanum insanum* (INS) and their hybrid (HYB) in the conditions tested (control, 200 mM and 400 mM of NaCl). Different lowercase letters within the same row indicate statistically significant differences between treatments, and different uppercase letters within the same column indicate statistically significant differences between genotypes for each measured compound (*p* < 0.05), according to the Student–Newman–Keuls multiple range test.

		Treatment (NaCl Concentrations)		
		Control	200 mM	400 mM
ChlA	MEL	2.8 ± 0.8 ^aA^	3.1 ± 1.7 ^aA^	2.2 ± 0.9 ^aA^
	HYB	2.7 ± 1.3 ^aA^	2.7 ± 1.4 ^aA^	4.4 ± 1.9 ^aB^
	INS	1.5 ± 0.9 ^aA^	2.9 ± 1.9 ^aA^	1.8 ± 1.1 ^aA^
ChlB	MEL	0.9 ± 1.4 ^aA^	1.6 ± 1.3 ^aA^	0.8 ± 0.9 ^aA^
	HYB	0.5 ± 0.4 ^aA^	0.3 ± 0.1 ^aA^	0.7 ± 0.5 ^aA^
	INS	1.0 ± 1.1 ^aA^	0.9 ± 0.3 ^aA^	0.4 ± 0.2 ^aA^
Caro	MEL	0.8 ± 0.4 ^aA^	0.9 ± 0.4 ^aA^	0.6 ± 0.4 ^aA^
	HYB	1.0 ± 0.3 ^aA^	0.6 ± 0.2 ^aA^	0.8 ± 0.3 ^aA^
	INS	0.9 ± 0.3 ^bA^	1.0 ± 0.2 ^bA^	0.5 ± 0.2 ^aA^

**Table 4:** Proline (Pro) (μmol g^−1^ dry weight), total soluble sugars (TSS) (mg equiv. glucose g^−1^ dry weight), malondialdehyde (MDA) (nmol g^−1^ dry weight), hydrogen peroxide (H_2_O_2_) (μmol g^−1^ dry weight), total phenolic compounds (TPC) (mg equiv. gallic acid g^−1^ dry weight), and total flavonoids (TF) (mg equiv. catechin g^−1^ dry weight) values (mean ± SD; n = 5) in *Solanum melongena* (MEL), *Solanum insanum* (INS) and their hybrid (HYB) in the conditions tested (control, 200 mM and 400 mM of NaCl). Different lowercase letters within the same row indicate statistically significant differences between treatments, and different uppercase letters within the same column indicate statistically significant differences between genotypes for each measured compound (*p* < 0.05), according to the Student–Newman–Keuls multiple range test.

		Treatment (NaCl Concentrations)		
		Control	200 mM	400 mM
Pro	MEL	11.9 ± 1.4 ^aA^	85.7 ± 13.9 ^bA^	134.1 ± 22.6 ^cA^
	HYB	107.8 ± 42.4 ^aB^	2155.9 ± 258.9 ^bB^	3217.5 ± 697.9 ^bB^
	INS	27.3 ± 35.5 ^aA^	2233.5 ± 265.5 ^bB^	3717.1 ± 318.1 ^cB^
TSS	MEL	160.2 ± 39.7 ^aA^	133.0 ± 63.7 ^aA^	224.1 ± 60.6 ^aA^
	HYB	205.8 ± 69.9 ^aA^	180.9 ± 50.1 ^aA^	209.9 ± 32.8 ^aA^
	INS	227.4 ± 120.0 ^aA^	144.6 ± 78.9 ^aA^	187.9 ± 31.4 ^aA^
MDA	MEL	623.7 ± 277.5 ^aA^	544.4 ± 119.8 ^aA^	493.9 ± 77.3 ^aA^
	HYB	658.3 ± 111.6 ^aA^	437.2 ± 147.5 ^aA^	443.3 ± 162.0 ^aA^
	INS	569.1 ± 323.2 ^aA^	597.3 ± 131.9 ^aA^	477.9 ± 171.3 ^aA^
H_2_O_2_	MEL	6.6 ± 0.9 ^aA^	5.6 ± 1.8 ^aA^	11.4 ± 7.33 ^aAB^
	HYB	5.5 ± 1.6 ^aA^	5.1 ± 2.8 ^aA^	9.4 ± 4.7 ^aA^
	INS	28.5 ± 6.7 ^aB^	21.3 ± 5.5 ^aB^	50.8 ± 11.8 ^bB^
TPC	MEL	23.3 ± 8.6 ^aA^	26.7 ± 9.6 ^aA^	36.2 ± 11.4 ^aA^
	HYB	37.8 ± 11.6 ^aB^	25.2 ± 3.3 ^aA^	32.5 ± 14.2 ^aA^
	INS	31.2 ± 6.5 ^aAB^	34.5 ±4.6 ^aA^	41.3 ± 11.3 ^aA^
	MEL	4.6 ± 3.5 ^aA^	7.8 ± 4.4 ^aA^	11.8 ± 6.6 ^aA^
TF	HYB	11.9 ± 4.2 ^aB^	11.3 ± 2.8 ^aAB^	9.7 ± 5.5 ^aA^
	INS	12.3 ± 1.5 ^aB^	15.2 ± 2.7 ^aB^	16.6 ± 5.1 ^aA^

**Table 5:** Superoxide dismutase (SOD), ascorbate peroxidase (APx) and glutathione reductase (GR) specific activities (units mg^−1^ protein) (mean ± SD; n = 5) in *Solanum melongena* (MEL), *Solanum insanum* (INS) and their hybrid (HYB) in the conditions tested (control, 200 mM and 400 mM of NaCl). Different lowercase letters within the same row indicate statistically significant differences between treatments, and different uppercase letters within the same column indicate statistically significant differences between genotypes for each measured compound (*p* < 0.05), according to the Student–Newman–Keuls multiple range test.

		Treatment (NaCl Concentrations)		
		Control	200 mM	400 mM
APx	MEL	48.9 ± 29.2 ^aA^	89.9 ± 58.2 ^aB^	87.4 ± 28.2 ^aA^
	HYB	98.5 ± 65.3 ^aA^	124.5 ± 20.7 ^aB^	267.2 ± 161.8 ^aA^
	INS	62.7 ± 64.0 ^aA^	16.7 ± 11.2 ^aA^	83.0 ± 28.6 ^aA^
SOD	MEL	90.2 ± 47.3 ^aA^	84.3 ± 34.9 ^aA^	89.2 ± 35.3 ^aAB^
	HYB	144.2 ± 55.0 ^aB^	92.3 ± 24.8 ^aA^	128.3 ± 34.4 ^aB^
	INS	61.9 ± 20.1 ^aA^	56.3 ± 13.1 ^aA^	55.2 ± 21.7 ^aA^
GR	MEL	948.1 ± 203.5 ^aA^	703.7 ± 196.8 ^aAB^	638.4 ± 68.6 ^aA^
	HYB	1018.9 ± 363.5 ^aA^	863.9 ± 369.0 ^aB^	1175.6 ± 438.7 ^aA^
	INS	349.1 ± 172.7 ^aA^	313.6 ± 157.0 ^aA^	359.7 ± 232.6 ^aA^

**Table 6:** Proline (Pro) values (μmol g^−1^ dry weight) (mean ± SD; n = 5) in adult plants of *Solanum melongena* (MEL), *Solanum insanum* (INS) and their hybrid (HYB) in control conditions and under long-term (eight weeks) 80 mM NaCl irrigation. Different lowercase letters within the same row indicate statistically significant differences between treatments, and different uppercase letters within the same column indicate statistically significant differences between genotypes (*p* < 0.05), according to the Student–Newman–Keuls multiple range test.

		Treatment (NaCl Concentrations)	
		Control	80 mM
Pro	MEL	13.6 ± 3.7 ^aA^	125.1 ± 54.1 ^bB^
	HYB	9.8 ± 6.1 ^aA^	46.5 ± 22.8 ^bA^
	INS	6.7 ± 4.8 ^aA^	18.6 ± 18.8 ^aA^

## Data Availability

Not applicable.
